# Changes in circulating cell-free nuclear DNA and mitochondrial DNA of patients with adolescent idiopathic scoliosis

**DOI:** 10.1186/s12891-019-2869-5

**Published:** 2019-10-25

**Authors:** Jiong Li, Longjie Wang, Guanteng Yang, Yunjia Wang, Chaofeng Guo, Shaohua Liu, Qile Gao, Hongqi Zhang

**Affiliations:** Department of Spine Surgery, Xiangya Hospital, Central South University, No. 87, Xiangya Road, Changsha, 410008 China

**Keywords:** Adolescent idiopathic scoliosis, Circulating cell-free nuclear DNA, Circulating cell-free mitochondrial DNA, Sex differences, Lenke types

## Abstract

**Background:**

Adolescent idiopathic scoliosis (AIS) which characterized by complex three-dimensional deformity of spine has been difficult to cure because of the unknown etiopathology and uncertainty of progression. Nowadays, circulating cell-free (ccf) DNA was found to be a potential biomarker for several benign and malignant diseases. However, whether ccf DNA can be a biomarker for AIS has not been reported yet. In this study, we investigate the circulating cell-free nuclear DNA (ccf n-DNA) and mitochondrial DNA (ccf mt-DNA) concentrations in the plasma of patients with AIS and controls (CT), and the changed plasma ccf n-DNA and ccf mt-DNA levels and their association with clinical parameters were assessed.

**Methods:**

The plasma of peripheral blood from 69 AIS patients and 21 age-matched CT was collected for ccf DNA analysis. Quantitative PCR was used to detect ccf n-DNA and ccf mt-DNA levels, and correlation analyses between the ccf n-DNA and ccf mt-DNA levels and clinical characteristics were conducted. Receiver operator curves (ROC) were used to analyze the sensitivity and specificity of ccf n-DNA and ccf mt-DNA levels to different characteristics.

**Results:**

The plasma ccf n-DNA levels of both *GAPDH* and *ACTB* were significantly decreased in AIS patients compared with those in controls, while the plasma ccf mt-DNA levels did not changed. According to sex-related analyses, the ccf n-DNA levels in male CT-M was higher than that in female CT and male AIS, but the ccf n-DNA levels in female AIS was not significantly changed when compared with male AIS or female CT. However, the concentration of ccf mt-DNA in female AIS increased significantly when compared with male AIS. Surprisingly, Lenke type-related analyses suggested that Lenke type 1 patients had lower ccf n-DNA levels, whereas Lenke type 5 patients had higher ccf mt-DNA levels compared with those of controls. However, a lower sensitivity and specificity of AIS predicted by ccf n-DNA or ccf mt-DNA levels was observed, whether in total, by sex, or by Lenke type.

**Conclusion:**

Although with no/little predictive accuracy of AIS/progressed AIS by ccf DNA levels, significantly changed plasma ccf DNA levels were observed in AIS patients compared with those in controls.

## Introduction

Adolescent idiopathic scoliosis (AIS) is a complex spinal deformity disease with a prevalence of 0.47–5.2% worldwide [1]. With the progression of AIS, the spine as well as the internal organs of the patients are severely affected. The curve progression rate in girls with AIS is higher than that in boys with AIS, and epidemiological studies showed that the female to male ratio rises from 1.4:1 to 7.2:1 as the curve progressed [1, 2]. Meanwhile, estrogen receptor gene polymorphism has been reported to be associated with AIS in various populations [3–6]. Estrogen was found to increase the incidence of scoliosis and curve severity in the pubescent bipedal rat scoliosis animal model [7]. These results suggest that sex influences the development and progression of AIS.

Six different curve types of AIS based on the identification of the major curve and the structural characteristics of the minor curves were presented according to the Lenke classification system, which provided a less than perfect but more reliable tool to assist surgeons with the choice of spinal fixation methods [8]. Lenke type 1 is a main thoracic curve; type 2 is a double thoracic curve with a proximal minor structural curve and a main thoracic major curve; type 3 is also a double major curve with the main thoracic curve being the major curve; type 4 is a triple major curve with structural curves in the thoracic region, major thoracic curve and curves in the thoracolumbar/lumbar region; type 5 is a single structural curve in the thoracolumbar/ lumbar region surrounded by two minor nonstructural curves; and type 6 is a thoracolumbar/lumbar-main thoracic double curve [8, 9]. The curve type has an impact on the progression of AIS because double curves progress more than single curves, and the least progression is in the curves in the lumbar region [2].

Given that the etiology of AIS is unclear, the treatment has focused on surgical prevention rather than causes aimed at curing, which would be more effective. However, the surgeons’ surgical approaches and fusion levels were also highly difficult to decide because of its complexity as well as uncertainty of progression. Although Waller, T. et al showed that an artificial neural network based on the expression levels of ACTB and GAPDH in different tissues of scoliotic patients might be used to differentiate familial from sporadic cases of idiopathic scoliosis [10], additional tools may still be needed for progressed and non-progressed AIS classification as well as disclosing the etiopathology of AIS.

Recently, circulating cell-free (ccf) DNA such as ccf nuclear DNA (n-DNA) and ccf mitochondrial DNA (mt-DNA) was found to be a potential biomarker for several benign and malignant diseases [11–15]. In addition, noninvasive detection of several fetal genetic aberrations can be conducted because the fetal genome circulates in maternal blood in the form of circulating DNA [16, 17]. It has been demonstrated that ccf DNA is not only produced by apoptosis and necrosis processes but also derived from active cellular release mechanisms shared by all organisms and cell types [18]. Additionally, ccf DNA can act as an intercellular messenger and even plays roles in the shaping of genomes and ultimately the process of evolution [18]. Moreover, other circulating nucleic acids like microRNAs (miRNAs) was shown to be a unique biomarker signature that diagnoses AIS with high sensitivity and specificity [19]. And Liu, X.Y. et al found abnormal expression of long noncoding RNAs (lncRNAs) in peripheral blood of AIS patients, and the expression of some lncRNAs was related to age, height, classification *etc* [20]. However, whether ccf DNA has been changed as well as miRNAs and lincRNAs in AIS patients is still undiscovered. In this study, the ccf n-DNA and ccf mt-DNA levels in the plasma of AIS patients and the control group were measured and quantified. This is the first attempt to find an association between ccf DNA and AIS.

## Methods

### Patients and controls

The plasma from 69 patients with AIS and 21 controls aged between 10 and 18 years were randomly collected carefully in Xiangya Hospital between 2016 and 2018 (Table 1 and Additional file 1). The patients were identified based on their clinical manifestations, X-ray, CT and MRI results, etc. And the controls included lumbar disc herniation patients and participants that were strictly screened with Adams’ forward bend test. The exclusion criteria of the patients and controls were as follows: individuals with neuromuscular or genetic disease; and individuals using hormones or immune inhibitors. This study has been conducted adhering to the principles of the Declaration of Helsinki II and approved by the medical ethics committee of Xiangya Hospital, Central South University (ethical code: 201703358).

**Table 1:** Demographic of study populations

Characteristics		CT (*N* = 21)		AIS (*N* = 69)		*P* value
		Male	Female	Male	Female	
Case NO		6	15	17	52	0.777
Age (years)		13.17 ± 1.05	14.40 ± 0.75	14.47 ± 0.39	14.27 ± 0.25	0.599^a^
						0.590^b^
Major curve Cobb Angle (^o^)		–	–	36.76 ± 4.51	35.40 ± 2.09	0.762
Lenke types	1	–	–	11	29	–
	5	–	–	4	20	–
	2	–	–	1	1	–
	3	–	–	0	1	–
	4	–	–	1	1	–
	6	–	–	0	0	–

*CT* control group, *AIS* adolescent idiopathic scoliosis group, *P*^a^ comparison of CT and AIS, *P*^b^ comparison of CT-M, *CT-F* AIS-M and AIS-F

### Extraction of ccf DNA in plasma

The plasma samples were collected by centrifuging blood at 4 °C, 2000 g for 10 min, and stored at − 80 °C until extraction of ccf DNA. The ccf DNA in the plasma was extracted following the manufacturers instruction of Circulating Nucleic Acid Kit (CWbio, Beijing, China). Briefly, mixed 50 μl proteinase K, 500 μl plasma and 400 μl buffer CL was incubated at 60 °C for 30 min, and then 360 μl Buffer CB was added in to the mixture before which was passed through the Spin Columns DF. 500 μl Buffer GW1, 750 μl Buffer GW2 and 750 μl ethanol were used to wash the Spin Columns DF combined with ccf DNA successively. The ccf DNA was eluted by 30 μl Buffer EBL and stored at − 20 °C for use.

### Real time PCR (qPCR)

The qPCR was performed as previous with specific primers for the nucleic genes and mitochondrial genes respectively (Table 2) [21, 22]. The concentration of ccf n-DNA in plasma were expressed as genome equivalent/ml according to the qPCR results of Glyceraldehyde 3-phosphate dehydrogenase (*GAPDH*) and beta-actin (*ACTB*). The concentration of ccf mt-DNA was obtained by calculating the ΔCT of n-DNA and mt-DNA (CT_GAPDH_ -CT_MT-ND1_) in the same sample as the length of mitochondrially encoded NADH dehydrogenase 1 (*MT-ND1*) sequence was similar to *GAPDH*, and used as exponent of two (2ΔCT).

**Table 2:** Primers sequences for cell free nuclear DNA and mitochondrial DNA

GENE	Primer sequence (5′–3′)	Length (bp)
*GAPDH*	F: GGAGAAGCTGAGTCATGGGT	219
	R: AAGACGGAATGGGGAGAAGG	
*ACTB*	F: CTGGAACGGTGAAGGTGACA	65
	R: CGGCCACATTGTGAACTTTG	
*MT-ND1*	F: CCCTAAAACCCGCCACATCT	214
	R: CCGATCAGGGCGTAGTTTGA	

Abbreviation: *F* Forward, *R* Reverse

### Statistical analysis

All results were expressed as mean ± SEM (standard error of the mean). Statistical significance was determined by student *t*-test or the one-way ANOVA, followed by post hoc t-test with LSD correction for multiple comparison. The sex difference was calculated by *x*^2^ test. The Pearson correlation coefficient was applied for the correlation analysis. A receiver operating characteristic (ROC) curve was used to determine the ccf n-DNA or ccf mt-DNA concentration for distinguishing different groups, and the best cut-off value was determined by maximum Youden index. Differences were considered statistically significant when *p* < 0.05. All statistical analyses were performed by using the SPSS 22.0 (SPSS Inc., Chicago, IL, USA).

## Results

### General physiological features of patients with AIS and the control group

A total of 90 plasma samples from 21 controls and 69 AIS patients aged between 10 and 18 years (y) were investigated in this study. As shown in Table 1, there were 6 male and 15 female controls (CT) and 17 male and 52 female patients with AIS, and the mean age of those in the four groups was 13.17 y for CT males (CT-M), 14.41 y for CT females (CT-F), 14.47 y for AIS males (AIS-M), and 14.28 y for AIS females (AIS-F). Neither an age difference nor a sex difference was observed between controls and AIS patients (Table 1 and Additional file 1). The degree of the major curve Cobb angle in the AIS-M and AIS-F groups was 36.76 and 35.40, respectively, with no significant change. In addition, the AIS-M group consisted of 11 Lenke type 1 (L1), 4 Lenke type 5 (L5), 1 Lenke type 2 (L2), 0 Lenke type 3 (L3), 1 Lenke type 4 (L4), and 0 Lenke type 6 (L6) patients, whereas the AIS-F group had 29 L1, 20 L5, 1 L2, 1 L3, 1 L4 and 0 L6 patients.

### Alteration of plasma ccf n-DNA and ccf mt-DNA levels in AIS patients compared with those in controls

**Fig. 1:**
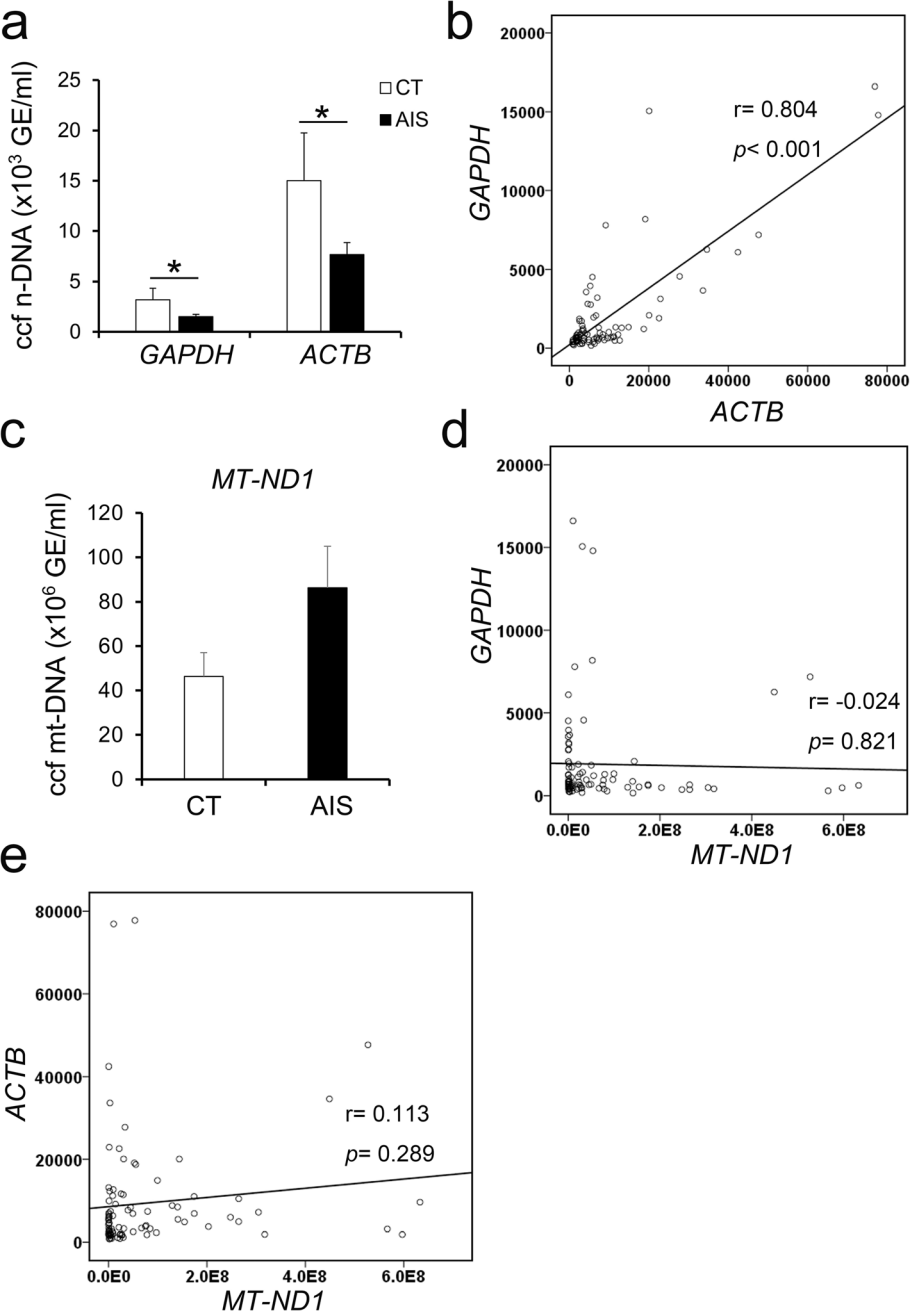
Changes of plasma ccf n-DNA and ccf mt-DNA levels in AIS patients compared with controls. **a**, Concentration of plasma ccf n-DNA in AIS patients and controls; **b**, Correlation of ccf n-DNA-GAPDH and ccf n-DNA-ACTB; **c**, Concentration of plasma ccf mt-DNA in AIS patients and controls; **d**, Correlation of ccf n-DNA-GAPDH and ccf mt-DNA; **e**, Correlation of ccf n-DNA-ACTB and ccf mt-DNA. *n* = 21–69, *p < 0.05

Two housekeeping genes, namely, *GAPDH* and *ACTB*, were used for ccf n-DNA analysis. As shown in Fig. 1a, compared with those in controls, significantly decreased plasma ccf n-DNA levels were observed in AIS patients according to the qPCR results for *GAPDH* (AIS, 1517 ± 223 GE/ml vs CT, 3185 ± 1145 GE/ml, *p* = 0.027). Similarly, the ccf n-DNA concentration of AIS patients according to the qPCR results for *ACTB* was also significantly lower than controls (AIS, 7689 ± 1159 GE/ml vs CT, 15017 ± 4737 GE/ml, *p* = 0.030). Although a significantly higher concentration of plasma ccf n-DNA-ACTB than ccf n-DNA-GAPDH was observed in both AIS patients and controls, a positive correlation between the levels of ccf n-DNA-ACTB and ccf n-DNA-GAPDH was observed (Fig. 1a. b and Additional file 2). These data could result from the phenomenon whereby the predominant n-DNA molecules in plasma were shorter than 200 bp [23].

In addition, the mitochondrial genes of *MT-ND1* were used for ccf mt-DNA analysis. Surprisingly, the concentration of ccf mt-DNA between AIS patients and controls was not significantly changed (AIS, 86372,782 ± 18,634,320 GE/ml vs CT, 46,273,967 ± 10,767,971 GE/ml, *p* = 0.066) (Fig. 1c and Additional file 2). The concentration of plasma ccf mt-DNA was much higher than that of the ccf n-DNA in both AIS patients and controls (Fig. 1c). However, no correlation between ccf n-DNA (*GAPDH* or *ACTB*) and ccf mt-DNA was observed (Fig. 1d, e).

**Fig. 2:**
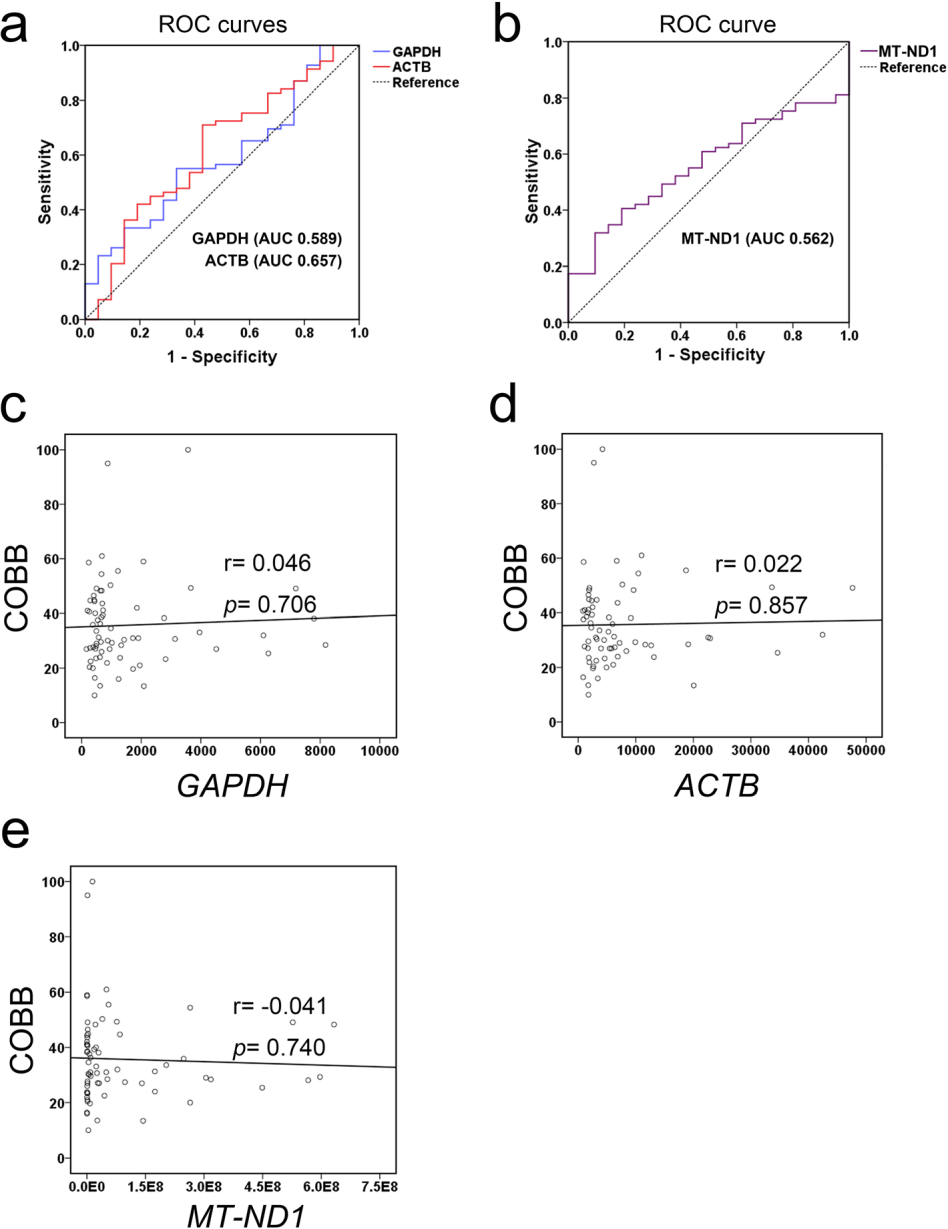
Ccf DNA analysis in AIS patients and controls. **a**, Receiver operative characteristics (ROC) curves for ccf n-DNA levels discriminating between AIS patients and controls; **b**, ROC curve for ccf mt-DNA levels discriminating between AIS patients and controls; **c**-**e**, Correlation between the plasma ccf n-DNA-GAPDH, ccf n-DNA-ACTB, ccf mt-DNA concentration and the major curve Cobb angle in AIS patients. *n* = 21–69

To evaluate the predictive capability of ccf n-DNA and ccf mt-DNA in AIS, we performed ROC analysis. The best cutoffs were 732 GE/ml for ccf n-DNA-GAPDH, 6953 GE/ml for ccf n-DNA-ACTB, and 1,684,972 GE/ml for ccf mt-DNA based on the areas under the curve (AUCs) of 0.589 (95% CI, 0.456 to 0.722), 0.657 (95% CI, 0.525 to 0.789), and 0.562 (95% CI, 0.440 to 0.683), respectively (Fig. 2a, b and Table 3). As shown in Table 3, the concentration of ccf n-DNA-GAPDH and ccf mt-DNA had no predictive capability, while the ccf n-DNA-ACTB concentration showed a low predictive accuracy. Moreover, Pearson analyses suggested no correlation between the major curve Cobb angle and ccf n-DNA (ccf n-DNA-GAPDH and ccf n-DNA-ACTB) or ccf mt-DNA (Fig. 2c-e).

**Table 3:** ROC curves comparing the ccf n-DNA and mt-DNA to discriminate AIS patients and controls

Test	Cutoff (GE/ml)	AUC (95% CI)	Sensitivity	Specificity	*P* value
n-DNA-GAPDH	732	0.589 (0.456–0.722)	0.551	0.667	0.217
n-DNA-ACTB	6953	0.657 (0.525–0.789)	0.710	0.619	0.030
mt-DNA	1,684,972	0.562 (0.440–0.683)	0.319	0.905	0.393

Abbreviation: *ccf* cell free circulating, *AUC* area under curves

These data may suggest that AIS patients have lower ccf n-DNA levels than controls, although little predictive accuracy of the ccf DNA levels was found. The ccf n-DNA (ccf n-DNA-GAPDH and ccf n-DNA-ACTB) or the ccf mt-DNA could not discriminate the major curve Cobb angle-related progression of AIS.

### Association of sex with the different plasma ccf n-DNA and ccf mt-DNA levels in AIS patients and controls

**Fig. 3:**
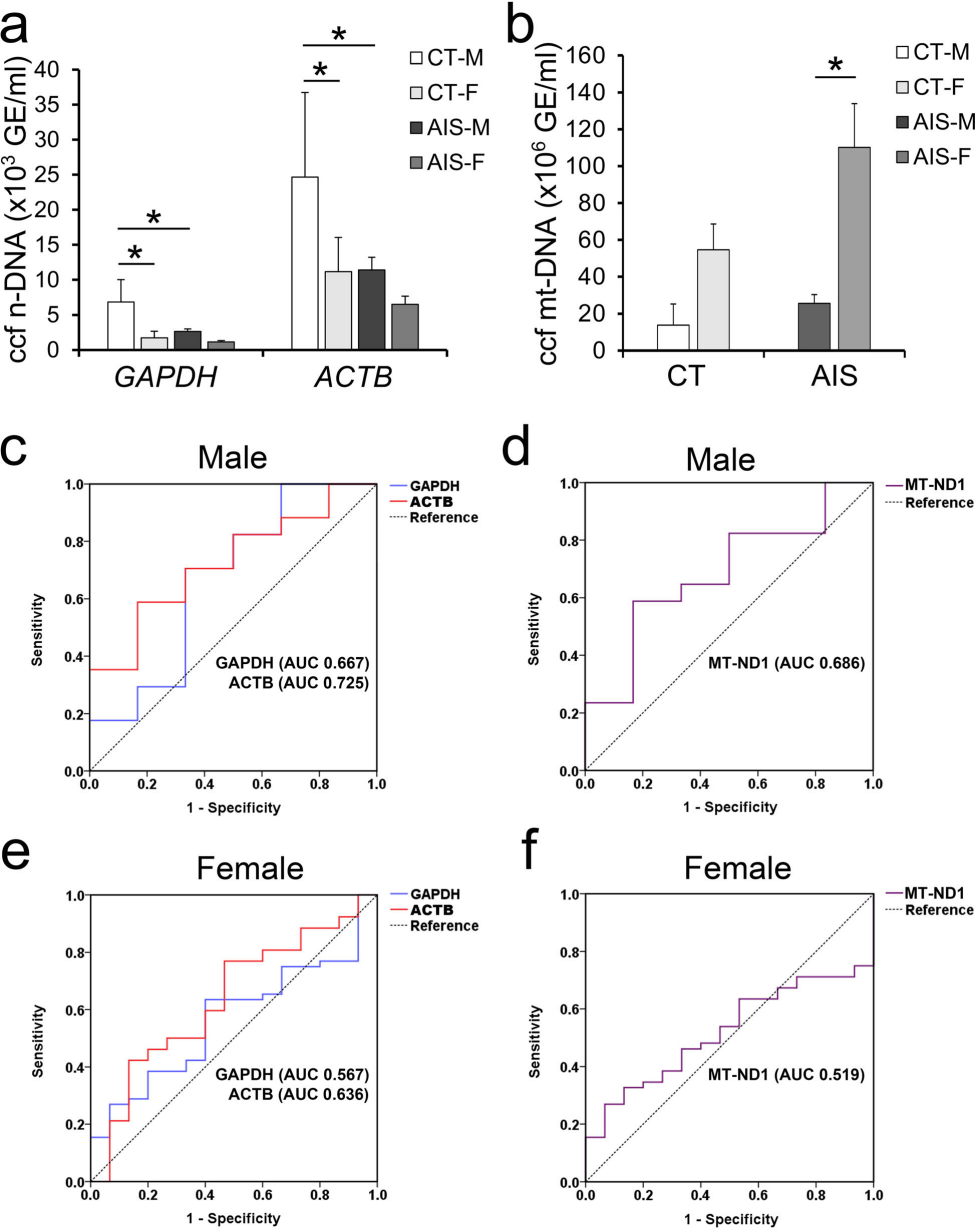
Gender differences of plasma ccf DNA levels in AIS patients and controls. **a**, Concentration of plasma ccf n-DNA in AIS patients and controls by gender; **b**, Concentration of plasma ccf mt-DNA in AIS patients and controls by gender; **c**, **d**, ROC curves for ccf n-DNA and mt-DNA levels discriminating between AIS patients and controls in males; **e**, **f**, ROC curves for ccf n-DNA and mt-DNA levels discriminating between AIS patients and controls in females. CT-M, male controls; CT-F, female controls; AIS-M, male AIS patients; AIS-F, female AIS patients. *p < 0.05

Because the influence of sex was suggested to play a role in the development and progression of AIS, and because the effect of sex on the ccf n-DNA and ccf mt-DNA levels in AIS patients and age-matched controls was unknown, we further analyzed the plasma ccf n-DNA and ccf mt-DNA levels by sex. As shown in Fig. 3a, the concentration of ccf n-DNA-GAPDH in CT-M was higher than that in CT-F (CT-M, 6824 ± 2916 GE/ml vs CT-F, 1730 ± 937 GE/ml, *p* < 0.001), while the ccf n-DNA-GAPDH concentration between AIS-M and AIS-F was not significantly changed (AIS-M, 2631 ± 620 GE/ml vs AIS-F, 1153 ± 196 GE/ml, *p* = 0.057). Additionally, the ccf n-DNA-GAPDH concentration of AIS-M was significantly decreased compared with that of CT-M (*P* = 0.002), while no difference between the ccf n-DNA-GAPDH concentration of AIS-F and CT-F was observed (*P* = 0.474) (Fig. 3a). And similar changes of ccf n-DNA-ACTB concentration were found between groups (CT-M, 24640 ± 12,105 GE/ml vs CT-F, 11168 ± 4871 GE/ml, *p* = 0.028; AIS-M, 11,414 ± 2940 GE/ml vs AIS-F, 6471 ± 1172 GE/ml, *p* = 0.180; CT-M vs AIS-M, *p* = 0.036; CT-F vs AIS-F, *p* = 0.297). On the other hand, the concentration of ccf mt-DNA was not significantly changed between any of the two groups except that the markedly increased ccf mt-DNA concentration of AIS-F was indicated compared with that of AIS-M (CT-M, 25,432,621 ± 11,440,608 GE/ml vs CT-F, 54,610,505 ± 13,993,774 GE/ml, *p* = 0.653; AIS-M, 13,753,634 ± 4,870,270 GE/ml vs AIS-F, 110,113,658 ± 23,812,914 GE/ml, *p* = 0.012; CT-M vs AIS-M, *p* = 0.837; CT-F vs AIS-F, *p* = 0.252) (Fig. 3b).

Similarly, the ROC analyses were conducted by sex. The best cutoffs calculated by ROC curves between CT-M and AIS-M were 3170 GE/ml for ccf n-DNA-GAPDH, 6940 GE/ml for ccf n-DNA-ACTB, and 2,178,401 GE/ml for ccf mt-DNA, based on the AUCs of 0.667 (95% CI, 0.384 to 0.950), 0.725 (95% CI, 0.503 to 0.948), and 0.686 (95% CI, 0.443 to 0.930), respectively (Fig. 3c, d and Table 4). The best cutoffs calculated by ROC curves between CT-F and AIS-F were 732 GE/ml for ccf n-DNA-GAPDH, 7291 GE/ml for ccf n-DNA-ACTB, and 1,684,972 GE/ml for ccf mt-DNA, based on the AUCs of 0.567 (95% CI, 0.417 to 0.716), 0.636 (95% CI, 0.476 to 0.796), and 0.519 (95% CI, 0.379 to 0.660), respectively (Fig. 3e, f and Table 4).

**Table 4:** ROC curves comparing the ccf n-DNA and mt-DNA to discriminate AIS patients and controls by gender

Test		Cutoff (GE/ml)	AUC (95% CI)	Sensitivity	Specificity	*P* value
Male	n-DNA-GAPDH	3170	0.667 (0.384–0.950)	0.706	0.667	0.234
	n-DNA-ACTB	6940	0.725 (0.503–0.948)	0.588	0.833	0.107
	mt-DNA	2,178,401	0.686 (0.443–0.930)	0.588	0.833	0.183
Female	n-DNA-GAPDH	732	0.567 (0.417–0.716)	0.635	0.600	0.434
	n-DNA-ACTB	7291	0.636 (0.476–0.796)	0.769	0.533	0.111
	mt-DNA	1,684,972	0.519 (0.379–0.660)	0.269	0.933	0.821

Abbreviation: *ccf* cell free circulating, *AUC* area under curves

### Association of the Lenke type with different plasma ccf n-DNA and ccf mt-DNA levels in AIS patients and controls

**Fig. 4:**
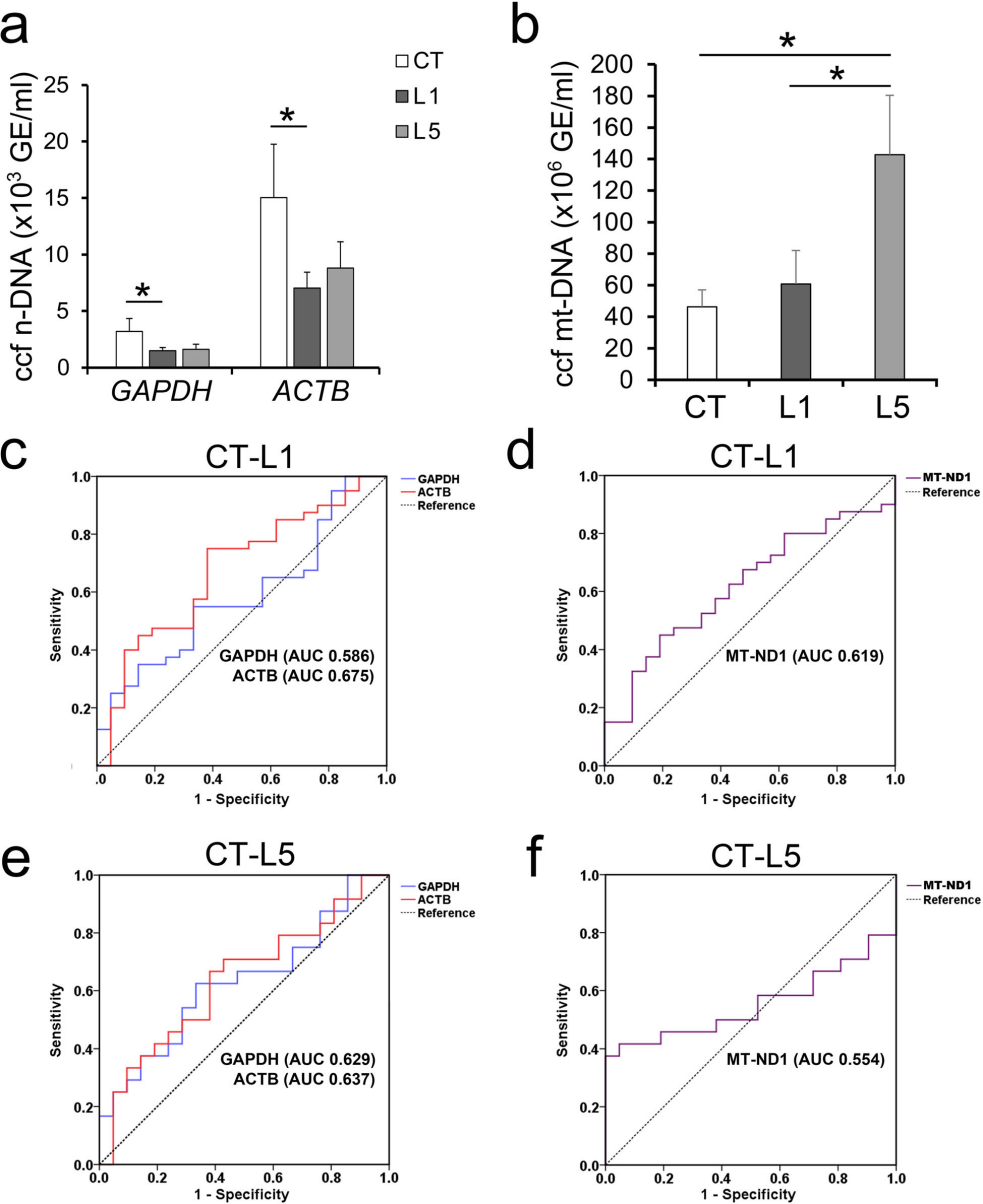
Lenke types differences of plasma ccf DNA levels in AIS patients and controls. **a**, Concentration of plasma ccf n-DNA in AIS patients and controls by Lenke types; **b**, Concentration of plasma ccf mt-DNA in AIS patients and controls by Lenke types; **c**, **d**, ROC curves for ccf n-DNA and mt-DNA levels discriminating between L1 patients and controls; **e**, **f**, ROC curves for ccf n-DNA and mt-DNA levels discriminating between L5 patients and controls. *p < 0.05

Given that the curve type also has an impact on the progression of AIS, we divided the AIS group into more groups according to the Lenke types. Since included Lenke 2–4 patients were rare, only L1 and L5 were further analyzed. Surprisingly, Lenke type-related differences in ccf DNA levels were observed in our study. As shown in Fig. 4a, compared with controls, L1 was decreased in the concentration of ccf n-DNA-GAPDH, while the L5 had no significant changes (L1, 1502 ± 271 GE/ml, *p* = 0.046; L5, 1618 ± 462 GE/ml, *p* = 0.093). Meanwhile, an obvious decrease in the ccf n-DNA-ACTB concentration was observed only in L1 when compared with controls (L1, 7030 ± 1404 GE/ml, *p* = 0.047; L5, 8806 ± 2334 GE/ml, *p* = 0.169) (Fig. 4a). No changes in either the ccf n-DNA-GAPDH or ccf n-DNA-ACTB concentration were observed between the two Lenke groups (Fig. 4a). Nevertheless, compared with controls, the concentration of ccf mt-DNA was significantly increased in L5 rather than in L1 (L1, 60,730,116 ± 21,391,393 GE/ml, *p* = 0.703; L5, 142,705,485 ± 37,810,843 GE/ml, *p* = 0.024) (Fig. 4b). Additionally, the ccf mt-DNA concentration of L1 was lower than L5 (L1 vs L5, *p* = 0.026) (Fig. 4b).

The ROC curves between controls and L1 or L5 are shown in Fig. 4c-f. The best cutoffs between CT and L1 were 729 GE/ml for ccf n-DNA-GAPDH, 6940 GE/ml for ccf n-DNA-ACTB, and 4,089,116 GE/ml for ccf mt-DNA, based on the AUCs of 0.586 (95% CI, 0.438 to 0.733), 0.675 (95% CI, 0.532 to 0.818), and 0.619 (95% CI, 0.477 to 0.762), respectively (Fig. 4c, d and Table 5). The best cutoffs between CT and L5 were 732 GE/ml for ccf n-DNA-GAPDH, 6953 GE/ml for ccf n-DNA-ACTB, and 164,261,352 GE/ml for ccf mt-DNA, based on the AUCs of 0.629 (95% CI, 0.465 to 0.793), 0.637 (95% CI, 0.473 to 0.801), and 0.554 (95% CI, 0.376 to 0.731), respectively (Fig. 4e, f and Table 5). Similarly, only the ccf n-DNA-ACTB concentration showed a low predictive accuracy for L1, while the concentrations of ccf n-DNA-GAPDH and ccf mt-DNA had no predictive capability for any Lenke types.

**Table 5:** ROC curves comparing the ccf n-DNA and mt-DNA to discriminate controls and Lenke 1 or Lenke 5 AIS patients

Test		Cutoff (GE/ml)	AUC (95% CI)	Sensitivity	Specificity	*P* value
Lenke 1	n-DNA-GAPDH	729	0.586 (0.438–0.733)	0.667	0.550	0.274
	n-DNA-ACTB	6940	0.675 (0.532–0.818)	0.619	0.750	0.026
	mt-DNA	4,089,116	0.619 (0.477–0.762)	0.450	0.810	0.129
Lenke 5	n-DNA-GAPDH	732	0.629 (0.465–0.793)	0.625	0.667	0.139
	n-DNA-ACTB	6953	0.637 (0.473–0.801)	0.667	0.619	0.116
	mt-DNA	164,261,352	0.554 (0.376–0.731)	0.375	1.000	0.539

Abbreviation: *ccf* cell free circulating, *AUC* area under curves

Together, our data suggested that AIS patients had significantly lower ccf n-DNA levels than controls. Specifically, the AIS patients of L1 had significantly decreased ccf n-DNA levels, while the AIS patients of L5 had markedly increased ccf mt-DNA levels.

## Discussion

To date, the conventional options that are recommended for preventive treatment for AIS patients are observation, bracing, and surgery because of the unclear etiology. Surgery is the most common and most effective treatment for patients with curves indicating a high likelihood of progression. However, surgery also has high risk and high cost. To reveal the pathology and mechanism of AIS, it is important to explore better treatment options. On the other hand, if we could accurately predict the progression of AIS patients, it would greatly help to improve disease management and provide better treatment by anticipating or even preventing surgery.

In 1948, Mandel and M’etais first reported the presence of ccf DNA in human blood [24]. Although the origin of ccf DNA remains poorly understood so far, it has been investigated as a biomarker for monitoring disease onset and/or progression [25]. Most of the studies on ccf DNA have shown higher ccf n-DNA and ccf mt-DNA concentrations than controls in many diseases, which may be associated with the original theory of ccf DNA being released from apoptotic cells or necrotic cells [15, 26]. However, in our study, significantly lower plasma ccf n-DNA concentrations but no significantly changed ccf mt-DNA concentrations were observed in AIS patients than in controls (Fig. 1a and c). The inconsistency in the changes between ccf n-DNA and ccf mt-DNA levels and the uncorrelated ccf mt-DNA levels and ccf n-DNA levels in AIS as well as in certain diseases may imply that ccf mt-DNA plays unique pathophysiological roles distinct from ccf n-DNA (Fig. 1) [14, 25].

In addition to the apoptotic or necrotic fraction of ccf n-DNA, the actively released fraction of ccf n-DNA with intercellular messaging capabilities may contribute enormously to pathogenesis [27]. The decrease in ccf n-DNA levels, when functioning as an intercellular messenger in adolescents, may participate in the dysregulation of development in our AIS patients. Elevated levels of ccf mt-DNA are associated with differential metabolic profiles and conditions characterized by chronic inflammation or oxidative stress that are involved in the pathogenesis of muscle wasting disorders [26, 28]. Similarly, elevated oxidative stress was found in the paraspinal muscles of AIS patients compared with controls in our previous study [29]. And we assume that the ccf mt-DNA levels in plasma might be affected by the accumulated oxidative stress in the muscles of AIS patients. In addition, possible association between expression level of ACTB, GAPDH and familial history of idiopathic scoliosis was proved [10]. Therefore, although the ROC analyses showed little predictive accuracy of ccf n-DNA and ccf mt-DNA in AIS, the significant changes of the ccf n-DNA concentration between AIS and controls may give a hint of their roles. Nevertheless, further investigation of the exact function or origins of both ccf n-DNA and ccf mt-DNA in AIS patients is needed.

Zhong et al. found no significant differences in the concentration of ccf DNA between age-matched men and women (age ≥ 20 y) [30]. However, in AIS patients and age-matched controls, we observed that sex differences in plasma ccf DNA levels existed. These results may stem from the fact that only the pubescent population around 10–18 y was investigated in our study. Moreover, the sex differences we observed were inconsistent between ccf n-DNA and ccf mt-DNA levels. Adolescent females showed potentially lower ccf n-DNA but higher mt-DNA levels than adolescent males (Fig. 3). These changes may be linked to epidemiological studies showing that girls are more susceptible to idiopathic scoliosis. Surprisingly, differences in plasma ccf DNA levels in different Lenke types were also indicated in our study. L1 patients had the decreased ccf n-DNA levels, while L5 patients had the elevated ccf mt-DNA levels (Fig. 4). Because the L1 and L5 types are the two most common Lenke types, this finding may suggest the potential role of plasma ccf DNA levels in AIS. Unfortunately, ccf n-DNA and ccf mt-DNA levels still showed no predictive accuracy for AIS by sex or by Lenke type according to the ROC analyses, which may indicate that better designed studies such as using more matched cohorts, collecting blood samples under the same condition rather than random as far as possible were further needed.

## Conclusions

This study showed significantly decreased plasma ccf n-DNA concentrations in AIS patients compared with that in controls. In addition, L1 patients had lower ccf n-DNA levels than controls, while L5 patients had the highest ccf mt-DNA levels among the groups. Thus, these data may indicate the important but different roles of plasma ccf n-DNA and ccf mt-DNA levels in AIS, which would hopefully offer a new angle for further investigation in prevention and treatment of AIS.

## Supplementary information

**Additional file 1.** Information of AIS patients and controls, The gender, Age, Lenke type and Cobb angle of studied population.

**Additional file 2.** The data of ccf DNA levels in AIS patients and controls, The ccf n-DNA-ACTB, ccf n-DNA-GAPDH and ccf mt-DNA-ND1 concentration of studied population.

## Data Availability

All data generated or analysed during this study are included in this published article and its supplementary information files.

## Abbreviations

ACTB: Beta-actin
AIS: Adolescent idiopathic scoliosis
AIS-F: AIS-female
AIS-M: AIS-male
AUC: Area under the curve
ccf DNA: Circulating cell free DNA
CT: Controls
CT-F: CT-female
CT-M: CT-male
GAPDH: Glyceraldehyde 3-phosphate dehydrogenase
L1: Lenke type 1
L2: Lenke type 2
L3: Lenke type 3
L4: Lenke type 4
L5: Lenke type 5
L6: Lenke type 6
mt-DNA: Mitochondrial DNA
MT-ND1: Mitochondrially encoded NADH dehydrogenase 1
n-DNA: Nuclear DNA
ROC: Receiver operating characteristic
SEM: Standard error of the mean
y: Years
